# Prediction of postoperative complications of pediatric cataract patients using data mining

**DOI:** 10.1186/s12967-018-1758-2

**Published:** 2019-01-03

**Authors:** Kai Zhang, Xiyang Liu, Jiewei Jiang, Wangting Li, Shuai Wang, Lin Liu, Xiaojing Zhou, Liming Wang

**Affiliations:** 1 School of Computer Science and Technology, Xidian University, No.2 South Taibai Rd, Xi’an, 710071 China; 2 State Key Laboratory of Ophthalmology, Zhongshan Ophthalmic Center, Sun Yat-sen University, Guangzhou, 510060 China; 3 Institute of Software Engineering, Xidian University, Xi’an, 710071 China; 4 School of Software, Xidian University, Xi’an, 710071 China; 5 School of Computer Science, Northwestern Polytechnical University, Xi’an, 710072 China

**Keywords:** Random forest, Naïve Bayesian, Association rules mining, Genetic feature selection, Medical decision making system

## Abstract

**Background:**

The common treatment for pediatric cataracts is to replace the cloudy lens with an artificial one. However, patients may suffer complications (severe lens proliferation into the visual axis and abnormal high intraocular pressure; SLPVA and AHIP) within 1 year after surgery and factors causing these complications are unknown.

**Methods:**

Apriori algorithm is employed to find association rules related to complications. We use random forest (RF) and Naïve Bayesian (NB) to predict the complications with datasets preprocessed by SMOTE (synthetic minority oversampling technique). Genetic feature selection is exploited to find real features related to complications.

**Results:**

Average classification accuracies in three binary classification problems are over 75%. Second, the relationship between the classification performance and the number of random forest tree is studied. Results show except for gender and age at surgery (AS); other attributes are related to complications. Except for the secondary IOL placement, operation mode, AS and area of cataracts; other attributes are related to SLPVA. Except for the gender, operation mode, and laterality; other attributes are related to the AHIP. Next, the association rules related to the complications are mined out. Then additional 50 data were used to test the performance of RF and NB, both of then obtained the accuracies of over 65% for three classification problems. Finally, we developed a webserver to assist doctors.

**Conclusions:**

The postoperative complications of pediatric cataracts patients can be predicted. Then the factors related to the complications are found. Finally, the association rules that is about the complications can provide reference to doctors.

**Electronic supplementary material:**

The online version of this article (10.1186/s12967-018-1758-2) contains supplementary material, which is available to authorized users.

## Background

The big data era for medicine is coming. Traditional statistical methods cannot effectively discover hidden laws from numerous medical records, whereas data mining [1] and machine learning [2], the most promising techniques, can tackle this problem. To investigate the application of data mining and machine learning on clinical records, we focus on pediatric cataracts, which can cause children blindness [3]. We obtained the detailed diagnosis and treatment information of 321 patients from one of the largest pediatric cataract databases (CCPMOH) [4] and evaluated the disease development in patients.

For children under age of two, pediatric cataracts prevent the light from penetrating into eyes and also delay the development of optic nerve [5]. Moreover, it is difficult for both patients and their parents to realize that children are already developing this disease, therefore, subsequent treatment is usually unfortunately delayed. Even if treatment is thankfully given, some unexpected complications (severe lens proliferation into the visual axis and abnormal high intraocular pressure) still arise after such treatment of replacing cloudy lens with artificial ones. Worse still, why and how these complications turn up are unknown according to the present studies. This study aims to adopt some data mining and machine learning techniques to predict these complications automatically within 1 year after surgery and determine which factors are more related to the complications. Because the heterogeneity of different patients is common in many diseases [6–8] and datasets that cover this disease are rare, this problem is difficult to tackle. Doctors and researchers required to circumvent this problem.

Many achievements have been made regarding the application of data mining and machine learning in the field of medical records. For example, Dheeraj Raju et al. used random forest to explore the factors influencing pressure ulcers and built a predictive model that is useful for doctors to predict the complication of this disease with the data collected over time [9]. Sónia Pereira et al. used obstetric and pregnancy factors to predict the appropriate delivery type to provide service of high quality, and to decide how to take care of pregnant women and newborn babies [10]. Aljumah et al. collected data from WHO (World Health Organization) to study the effectiveness of different treatment types with consideration of the age factor [11]. The results show that younger patients should not take medicine immediately to avoid side effects and older patients must take medicine to relieve sickness. Somanchi et al. applied SVM (support vector machine) and logistic regression to predict cardiac arrest in the future with the data from medical records. The result obtained from these methods is better than the results of previous methods and tools [12]. All of the above examples show that data mining is a powerful tool to study the medical data and the application of data mining techniques will produce some desirable results.

Inspired by the above researches, we attempted to mine meaningful knowledge from medical records of pediatric cataracts patients and then developed a medical decision making system to help doctors with predicting the complications of pediatric cataract patients. To study which factors contribute to the complications, some pairs of discriminant association rules are mined out using Apriori algorithm with preprocessed dataset. The antecedents of the association rules are combinations of various factors. The consequents of these association rules are whether a patient will have complications or a specific type of complication. According to the characteristics of the dataset, we used a simple discretization method and SMOTE (synthetic minority oversampling technique) to preprocess the dataset. Subsequently the number of positive samples (samples with complications or specific complication) was close to the number of negative samples (samples without complication or specific complication). Then the random forest and naïve Bayes classifier were used to predict whether patients would be affected by complications of different levels. Experimental result shows that random forest and Naïve Bayesian achieve accuracies which are above 76% and 75% in three binary classification problems [whether a patient suffers from complications (severe lens proliferation into the visual axis (SLPVA) and abnormal high intraocular pressure (AHIP)]; whether a patient suffers from the SLPVA; whether a patient suffers from AHIP). Then the number of trees of the random forest is investigated to find the most suitable number of trees to optimize the performance of random forest. The genetic feature selection was employed to find out which factors exactly caused the complications. Besides, we used additional 50 data to test the performance of random forest and Naïve Bayesian classifier, and both of them reach 65% for three classification problems. Finally, we exploited the random forest and association rules mining in the present research to construct an automatic complication prediction system for pediatric cataract patients so that patients with different degrees of complication can be warned and treated earlier.

## Methods

The dataset used in the current research is from Zhongshan Ophthalmic Center, Sun Yat-sen University, which is the most professional ophthalmic hospital in China and has set up the state of art ophthalmology laboratory [13]. There are a total of 321 samples in the dataset, where 26 samples suffer from SLPVA and AHIP simultaneously; 69 patients only suffer from SLPVA; 84 patients only suffer from AHIP, and 194 patients have no complication after surgery. In addition, the detailed information about all the attributes and their possible values are shown in Table 1.

**Table 1:** The specification of attributes in dataset

NO. of Attribute (attribute name)	Values
1. Gender	Male, female
2. Secondary IOL placement	Yes, no (primary IOL placement)
3. Operation mode	Lens aspiration (I/A), lens aspiration with posterior continuous curvilinear capsulorhexis (I/A + PCCC), lens aspiration with posterior continuous curvilinear capsulorhexis and anterior vitrectomy (I/A + PCCC + A-Vit)
4. Laterality	Unilateral cataracts, bilateral cataracts
5. Age at surgery (AS)	1, 2, 3, 4, 5, 6, 7
6. Area of cataracts (AC)	Large, small
7. Density of cataracts (DC)	Dense, sloppy
8. Position of cataracts (PC)	Covering the central area of lens, not covering the central area of lens
9. Nystagmus	Yes, no
10. Microphthalmia	Yes, no
11. Microcornea	Yes, no
12. Persistent hyperplastic primary vitreous (PHPV)	Yes, no

**Fig. 1:**
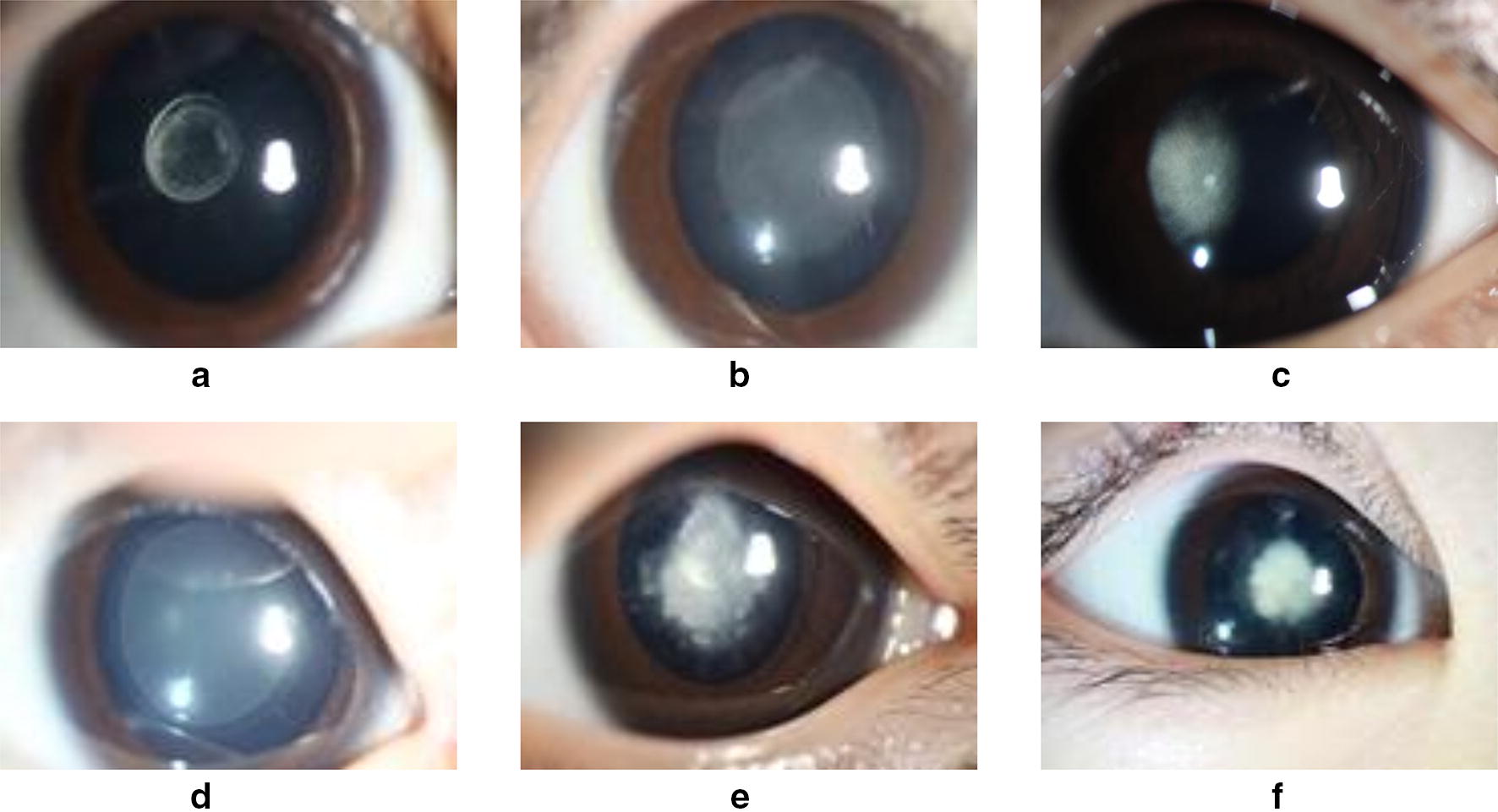
Three grading standards of severity of pediatric cataracts. **a** the area of cataract is small; **b** the area of cataract is large; **c** the density of the cataract is sloppy; **d** the density of the cataract is dense; **e** the cataract covers the central area of lens; **f** the cataract does not covering the central area of lens

Moreover, the sixth, seventh and eighth attributes in Table 1 are marked as the evaluation standards of the severity of pediatric cataracts, which are proposed by pediatric ophthalmologist with over 5 years of working experience in Zhongshan Ophthalmic Center. These three evaluation standards can portray the severity based on the morphology of the cataracts, where the density of the cataract can be classified as dense or sloppy, the spreading area of cataract can be expressed as big or small, and the relative position of the cataract can be assessed by whether focus covers the central area of the lens. Previous work of our team completed the grading (severity evaluation) of pediatric cataracts from these three aspects and made some progress [14], but the accuracy still cannot reach 100%. Therefore, we invite pediatric ophthalmologist with over 5 years of working experience to assess the three attributes of these samples in terms of the slit lamp images of patients. The difference among the three grading standards can be illustrated by Fig. 1.

In addition, according to relevant literature [15], the surgery age is particularly important in the development of the illness. Finally, according to the expert knowledge of three experienced ocular doctors, the operation age for all of the patients is discretized into seven sections: [0, 3], [4, 6], [7, 9], [10, 12], [13, 18], [19, 24], [> 24] (the unit is month) to facilitate classification. Except for the first and fifth attributes, the remaining attributes in Table 1 are recorded by doctors.

In current research, there are three main classification problems in this research: whether suffer from complications; whether suffer from SLPVA; whether suffer from AHIP. Therefore, the original dataset were decomposed into three sub datasets accordingly. Because of the classification problems in this research are non-numerical classification problems, random forest and Naïve Bayesian classifier which can tackle non-numerical classification problems were selected to complete classification tasks. Additionally, the mechanism of these two classification methods are pretty easy to understand and there are so many packages can be directly used to implement.

### Overview of methods

There are some common ways to tackle an imbalanced dataset, such as over sampling [16] (e.g., SMOTE [17]), under sampling [18] (e.g., clustering based under sampling [19]) and cost sensitive classification [20, 21]. Cost-sensitive method requires a cost matrix or a modified algorithm mechanism and architecture, which is more difficult for specific application. Therefore, SMOTE (Synthetic Minority Oversampling Technique) was adopted as an over sampling method to preprocess the dataset. Apriori algorithm and genetic feature selection were used to explore which combinations of factors are more likely to cause complication and which factors are more related with complications, respectively. Because the attributes in the dataset is non-numeric value, we choose Naïve Bayesian classifier and random forest to predict the postoperative complication of pediatric cataract patients.

### Apriori algorithm

Association rules mining [22] is a useful technology that is first used to analyze the shopping basket to find some shopping habits hidden in the shopping list of consumers and thus is used to promote sales. This method has been applied in many fields to discover the meaningful knowledge in specific problem, such as fault diagnosis of power transformers [22] and the detection of industrial intrusion [23].

The most commonly used algorithm to mine association rules is Apriori [23], which utilizes minimum support (the frequency of an itemset appeared in dataset for screening out the frequent item-set) to sift out the frequent *k*-item sets and then selects the association rules whose confidence is larger than the minimum confidence from frequent *k*-item set. In the first stage of the Apriori algorithm, with the assistance of a transcendental property that the nonempty subset of the frequent item set must be frequent, the Apriori algorithm finds the most common occurring mode (*k*-item set) whose support is larger than or equal to the minimum support that is set ahead until the (*k *+ 1)-item set produced from *k*-item set is empty; Then Apriori algorithm checks the confidence of every rule and retains only the rules with higher confidence. The confidence of an association rule is computed as Eq. (1).

1 $$Confidence(a \to b) = \frac{Frequency(a \cup b)}{Frequency(a)}$$

The association rules will come into being as many implications with the format “$a \to b$” using the Apriori algorithm, where *a* and *b* are the antecedents and consequents of association rules, respectively. The minimum support is set as 0 to let all items combine freely to produce association rules without considering their support and confidence. Because the aim is finding out the combinations of attributes which will or will not bring complication, the rules whose consequents are whether a patient suffers from complications (SLPVA and AHIP) are sifted out preliminarily. At last, the pairs of association rules with the same antecedent and different consequents will be sifted out again with Eq. (2), which means that the confidence of the pair of association rules should be far from each other mutually, where *threshold* is a parameter.

2 $$\frac{Confidence(a \to b)}{{Confidence(a \to b^{\prime})}} \ge Threshold, \quad b = \neg b^{\prime}, \quad b \in \{ 1,0\}$$

where, $a \to b$ and $a \to b^{\prime}$ are a pair of association rules with the same antecedent and different consequents. 1 and 0 refer to suffering from complications (SLPVA and AHIP) and not suffering from complication (SLPVA and AHIP), respectively. The association rules mining is widely applied in biomedical fields, such as microbial energy prospection [24], pollution epidemiology [25].

### SMOTE

SMOTE is a commonly used over-sampling technique for tackling imbalanced dataset. It used the *k*-nearest neighbor of each minority sample to produce more minority samples to offset the imbalance between majority class and minority class. The new minority samples is the linear combination of a minority sample and one of its nearest neighbor. Non-numerical minority data can also be preprocessed with SMOTE in a similar manner. In current research, we choose SMOTE as a comparison.

### Naive Bayesian classifier

Naive Bayes classifier [26–28] based on Bayes theorem and assumes all attributes are independent is a simple yet useful pattern recognition method. Given sample $(x,y)$ is a sample to be classified in multi-class classification problem, where $x = \left[ {x_{1} , \ldots ,x_{s} } \right]$, $y \in \left\{ {y_{1} , \ldots y_{o} } \right\}$, S and *o* are the attribute vector, label, the number of attributes and the number of classes, respectively. Naive Bayes classifier compute probabilities $P\left( {y_{1} \left| x \right.} \right), \ldots ,P\left( {y_{o} \left| x \right.} \right)$, and then classify the sample into the class with largest probability. These probabilities could be estimated with Bayes theorem [29, 30] which is shown as Eq. (3), then the label of a new sample can be computed with Eq. (4).

3 $$P\left( {y_{j} \left| x \right.} \right) = \frac{{P\left( {x\left| {y_{j} } \right.} \right)}}{P(x)}\quad j = 1, \ldots ,o$$

4 $$Label = \mathop {max}\limits_{j} p\left( {y_{j} \left| x \right.} \right)\quad j = 1, \ldots ,o$$

Because of the assumption that all attributes are independent and same denominator of Eq. (3) for all classes, the probabilities could be converted to be Eq. (5):

5 $$P\left( {x\left| {y_{j} } \right.} \right) = p(y_{j} ) = P\left( {x_{1} \left| {y_{j} } \right.} \right) \ldots P\left( {x_{S} \left| {y_{j} } \right.} \right)p(y_{j} ) = \prod\limits_{i = 1}^{S} P \left( {x_{i} \left| {y_{j} } \right.} \right)p(y_{j} )\quad j = 1, \ldots ,o$$

Naive Bayes classifier has been applied in a plenty of fields, such as detection of cardiovascular disease risk’s level [31], identification of hot spots in protein structures [32].

### Random forest

Random forest is an effective ensemble classifier that originates from decision tree, and this classifier can effectively avoid overfitting, which is a common issue with normal decision trees. Therefore, the decision tree is introduced first.

Decision tree [33, 34] is a type of classifier that regards the dataset to be an entire set, yet recursively divides this set into subsets as well according to a certain standard. During the latter process, all subsets are divided to the extent that they have no attributes to divide further or all samples in every subset belong to a uniform category. Decision tree has been applied in many fields, such as industrial safety [34], power [35] and financial behavior prediction [36].

There are three common standards to partition the dataset into subsets, namely, information gain, gain ratio and Gini index [37].

Random forest [38, 39] is an excellent ensemble learning algorithm that combines many decision trees with partial samples and partial attributes that are randomly selected from the whole dataset. Finally, the label of a testing sample is decided by voting in accordance with all decision trees of the random forest. Therefore, over-fitting problem can be avoided effectively. The current research adopted this algorithm to predict whether a patient will have complications and to classify the patients whether suffer from a specific complication. Random forest is also popular and widely applied in many disciplines, such as prediction of double-strand DNA breaks [40], localization of prostate cancer [39] and computational bioinformatics [41].

### Genetic feature selection

Next, genetic feature selection is used to find out which factors are more related with the complications of pediatric cataract patients. Genetic algorithm (GA) [42] can be used to select attributes that are useful for classification or more related to complications in the current research, where the fitness evaluation function of GA is the accuracy returned by classifier (random forest in this paper). In the flow of GA, the real factors which are associated with labels are searched out. The number of iteration steps and the size of population are 50 and 30, respectively. Genetic feature selection has been applied in many fields, such as image classification [43], text categorization [44], image feature extraction [45] and signal processing [46].

### Experimental settings and evaluation indicator

Because this classification problem (prediction of postoperative complication of pediatric cataracts patients) is a multi-label learning problem [47], common solution for this type of classification problem is to decompose the problem into several binary classification ones. At the same time, the dataset is imbalanced, so the samples of the minority class are fed into SMOTE to produce more minority samples. Consequently, there are three corresponding datasets for three binary classification problems (whether a patient suffers from complications; whether a patient suffers from SLPVA; whether a patient suffers from AHIP). The samples having complications (SLPVA and AHIP) are positive samples and the samples without complications (SLPVA and AHIP) are negative samples. Three-fold cross validation was adopted to permit a fair comparison of these methods.

All of the methods were implemented with MATLAB R2016a on a personal computer with an Intel 2.80 GHz i5 processor, 8G RAM. The objective of our study was to predict the complication of pediatric cataracts patients; the measures that were employed to evaluate the performance are shown as Eqs. (6–11):

6 $$Accuracy = \left( {TP + TN} \right)/\left( {P + N} \right)$$

7 $$Precision = TP/\left( {TP + FP} \right)$$

8 $$Sensitivity\;\left( {TPR,\;Recall} \right) = TP/\left( {TP + FN} \right)$$

9 $$FNR\;(false\;negative\;rate) = 1 - Sensitivity = FN/\left( {TP + FN} \right)$$

10 $$Specificity = TN/\left( {TN + FP} \right)$$

11 $$FNR\;(false\;positive\;rate) = 1 - Sensitivity = FP/\left( {TN + FP} \right)$$

where P and N are the number of positive samples and negative samples, respectively; TP indicates the number of positive samples classified into the positive class; FN denotes the number of positive samples classified as negative samples; TN is the number of negative samples recognized as negative samples; and FP refers to the number of negative samples identified as positive samples. In addition, the ROC (receiver operating characteristics) curve, which indicates how many positive samples are recognized conditioned on a given false positive rate and AUC (area under curve) which means the area of the zone under ROC curve are also adopted to assess the performance [48].

## Results

At first, we provide a part of the association rules mined out by Apriori algorithm in Additional file 1: Tables S1–S3. Then the postoperative complications of pediatric cataracts patients were predicted with random forest and Naïve Bayesian classifier. Then the contributing factors for the postoperative complications were found out with genetic feature selection. Finally, we used additional 50 data to verify the performance of random forest and Naïve Bayesian classifier, the accuracies of them are over 65% for three classification problems.

### Results of association rules mining

In order to find out the combination of factors which can cause complications with bigger probability, the Apriori algorithm is used to mine out the association rules about the complications.

### Classification performance of RF and NB

**Fig. 2:**
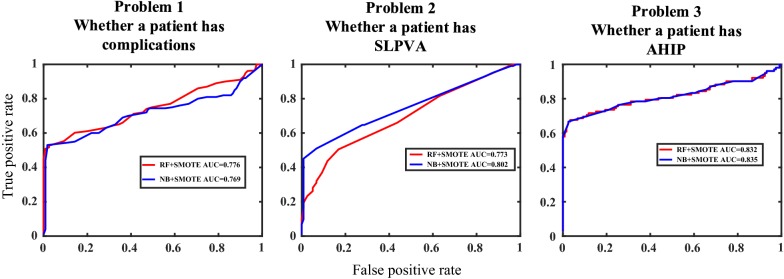
ROC curves and AUC values in three binary classification problem. (*ROC* receiver operating characteristics curve, *AUC*: area under curve, *RF* random forest; *SMOTE* synthetic minority oversampling technique, *NB* Naïve Bayesian classifier)

**Fig. 3:**
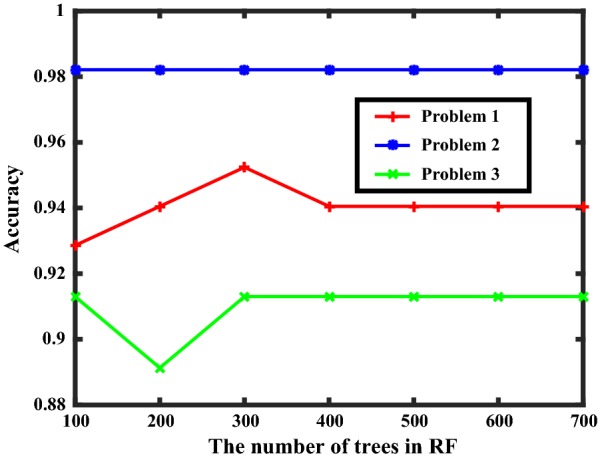
The relationship between accuracy and the number of trees of random forest. (RF means random forest)

The prediction of postoperative complication of pediatric cataracts patients is carried out with random forest and naïve Bayes classifier. The evaluation indicators for random forest and Naïve Bayes classifier with three different datasets that are preprocessed with SMOTE or original datasets in terms of three binary classification problems (whether a patient suffers from complications; whether a patient suffers from SLPVA; whether a patient suffers from AHIP) are shown in Table 2, where the format of these data is $\mu \pm \delta$ ($\mu$ is the mean value, $\delta$ is the standard deviation). The ROC curves combining AUC values in three binary classification problems with datasets preprocessed with SMOTE are shown in Fig. 2. The number of trees in random forest influences the classification performance and we investigate the relationship between them. Three classification problems were repeatedly solved with different number of trees and their accuracies are shown as Fig. 3.

**Table 2:** Performance indicators in three binary classification problems

Method		Accuracy	FNR	FPR
Problem 1: Whether a patient suffers from complications				
Random forest	–	0.757 ± 0.025	0.414 ± 0.031	0.128 ± 0.013
	SMOTE	0.762 ± 0.019	0.231 ± 0.013	0.220 ± 0.037
Naïve Bayesian	–	0.748 ± 0.025	0.465 ± 0.042	0.887 ± 0.023
	SMOTE	0.751 ± 0.032	0.270 ± 0.043	0.208 ± 0.044
Problem 2: Whether a patient suffers from SLPVA				
Random forest	–	0.810 ± 0.014	0.621 ± 0.089	0.071 ± 0.023
	SMOTE	0.753 ± 0.069	0.257 ± 0.054	0.258 ± 0.044
Naïve Bayesian	–	0.782 ± 0.014	0.155 ± 0.043	0.449 ± 0.100
	SMOTE	0.782 ± 0.043	0.244 ± 0.065	0.267 ± 0.025
Problem 3: Whether a patient suffers from AHIP				
Random forest	–	0.838 ± 0.024	0.580 ± 0.050	0.015 ± 0.014
	SMOTE	0.813 ± 0.016	0.228 ± 0.055	0.265 ± 0.025
Naïve Bayesian	–	0.847 ± 0.033	0 ± 0	0.321 ± 0.043
	SMOTE	0.816 ± 0.037	0.225 ± 0.047	0.265 ± 0.074

### Results of genetic feature selection

Next, we applied genetic algorithm (GA) combined with random forest to study which factors affect the complication, where the classification accuracy is used as the fitness of GA. Because GA is a type of probabilistic heuristic algorithm that involves random factors, the experiment was repeated ten times and the best result was selected as the final result. The experimental result shows that the accuracy for the first binary classification problem (whether suffers from complications) can reach 0.783 without using the gender and age at surgery (AS) attributes. The accuracy of the second binary classification (whether suffers from SLPVA) problem can reach 0.795 without the secondary IOL placement, operation mode, age at surgery and area of cataracts (AC) attributes. The accuracy of the third binary classification problem (whether suffer from AHIP) can reach 0.836 without gender, operation mode and laterality.

### Additional testing

**Fig. 4:**
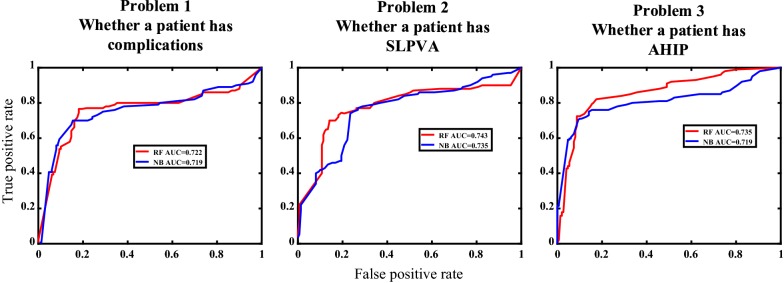
ROC curves and AUC values for additional testing

We used additional 50 samples to test performance of RF and NB, the accuracies reach 65% for three classification problems, respectively. The detailed information about additional testing is shown in Table 3. Figure 4 shows the ROC curves for additional testing. There are 27 samples do not suffer from complications; 18 samples suffer from SLPVA; 11 samples suffer from AHIP and five samples suffer both SLPVA and AHIP.

**Table 3:** The performance of RF and NB for additional testing

Problem	Algorithm	Accuracy	Sensitivity	Specificity
Whether a patient suffers from complications	Random forest	0.700	0.625	0.769
	Naïve Bayesian	0.700	0.731	0.667
Whether a patient suffers from SLPVA	Random forest	0.720	0.667	0.722
	Naïve Bayesian	0.660	0.611	0.688
Whether a patient suffers from AHIP	Random forest	0.700	0.636	0.718
	Naïve Bayesian	0.660	0.545	0.692

### Webserver of decision-making system

**Fig. 5:**
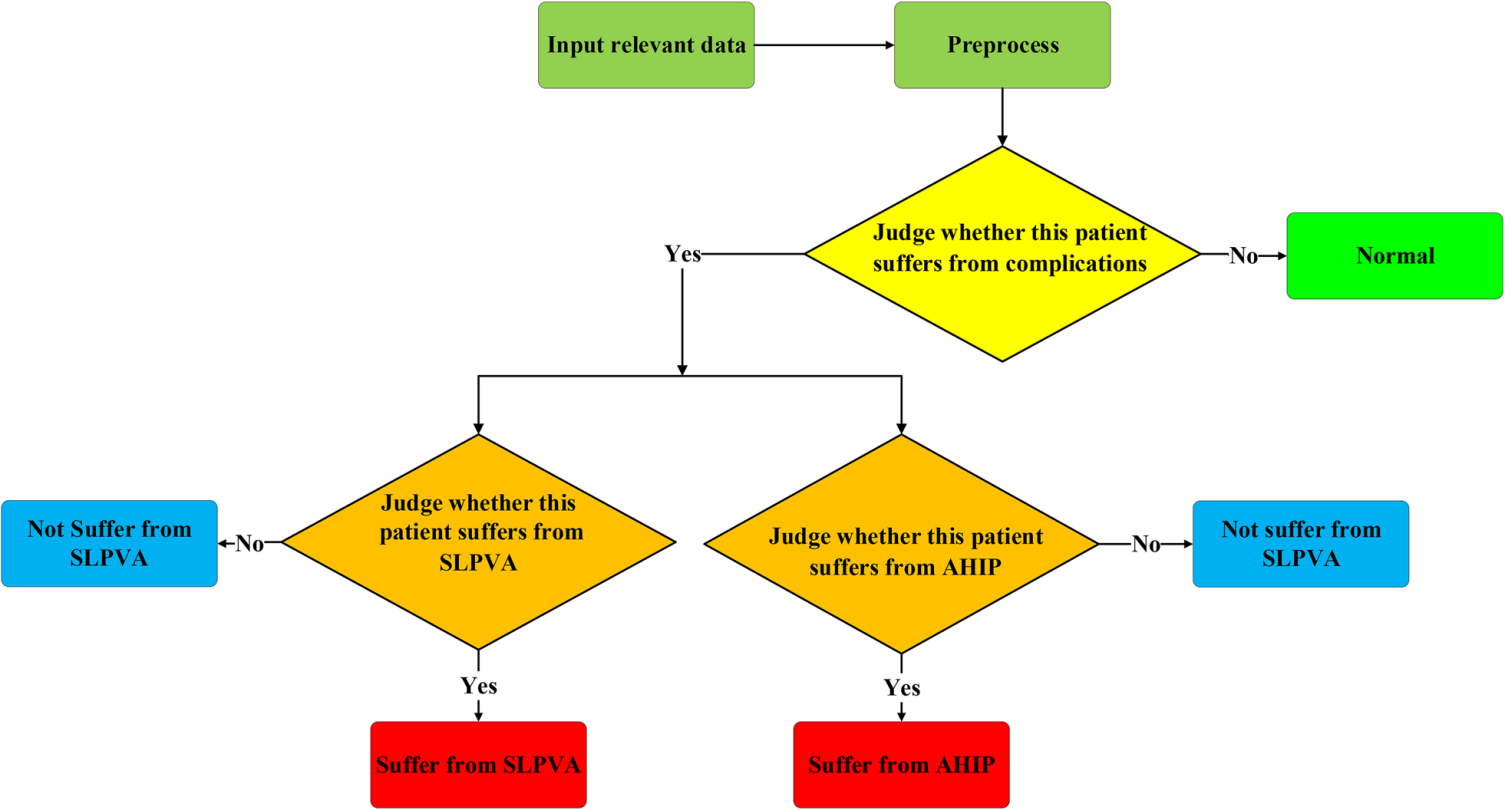
Flowchart of postoperative complication prediction. (At first, the inputted data is preprocessed with data discretion method. Then one of the three models is applied to distinguish whether a patient suffers from complication. If the patient is not normal, then the remaining two models are used to judge whether the patient has two types of complication. If the patient is judged to be normal, the remaining two models will not be used)

We developed and deployed a web based system to facilitate the prediction of complication levels for doctors and to show the association rules about complication. This system serves two functions: judging whether a patient suffers from complications or the specific type of complication using random forest as the flowchart shown in Fig. 5 and showing the association rules after user inputs parameter *threshold* for three types of problems. We provide two language versions (English and Chinese) for users and this web server is freely available at (English version) http://120.27.126.89:5001/option_xian (Chinese version) http://120.27.126.89:5001/option_xianch.

## Discussion

**Fig. 6:**
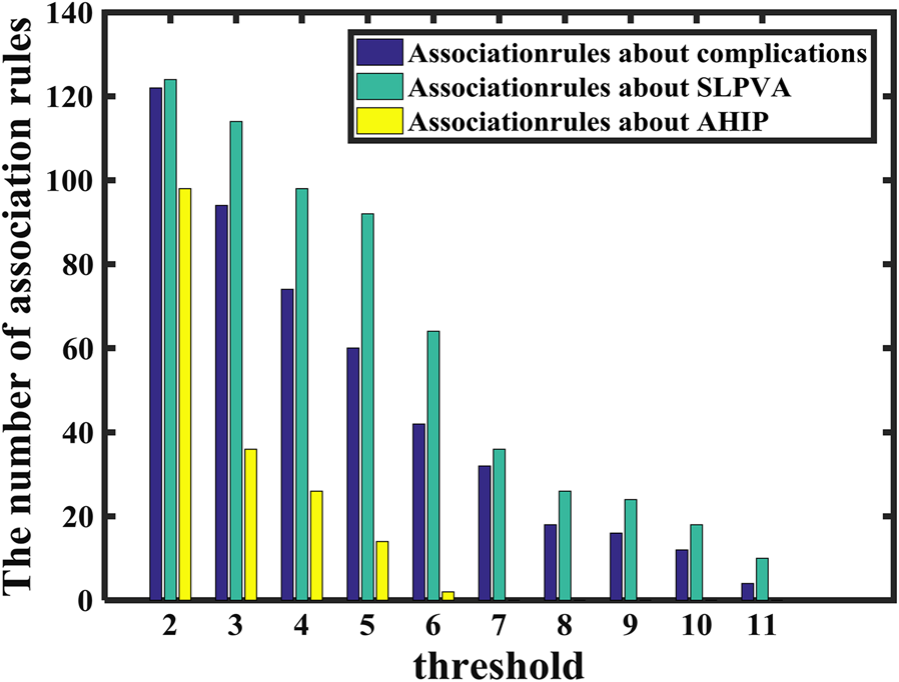
The relationship between *threshold* and the number of association rules

The number of association rules that are sifted out decreases while the *threshold* increases. The antecedents, consequents and confidences of all association rules are summarized to be three categories of tables, contributing as a part of experimental result. The reason why these steps are adopted is that we want to obtain some discriminant association rules that can provide some evidence about complications and whose consequences is different, but the reliability of these rules need to be verified with more clinical data. Some discriminant combinations of attributes (antecedent) can reflect the power of attributes for classification, such as secondary IOL replacement and gender used for classifying whether suffers from complications. However, these association rules need to be testified with more clinical data. The relationship between threshold and the number of associations rules is shown as Fig. 6.

These association rules can provide some reference for doctors to predict postoperative complication of pediatric cataracts. For example, a male patient whose age is between 13 and 18 months will be more unlikely to suffer from AHIP after the surgery on the basis of *threshold *= 6, which is shown in Additional file 1: Table S3.

SMOTE algorithm^17^ is a common over sampling method for imbalance dataset classification, it randomly sampling two samples from dataset and linearly combined them to be a new sample until imbalanced classes become balance. Here we used nominal SMOTE that is the extension of SMOTE in nonnumeric variables. All data were divided into multiple parts for cross-validation and then were preprocessed by SMOTE. Validation dataset is not preprocessed with SMOTE.

Sensitivity and specificity can be computed with FNR and FPR, so values for some sensitivity and specificity indices are not shown. We also use original dataset to perform these classification problems. SMOTE can effectively improve the classification performance. The classification performance with original dataset is so imbalanced, while SMOTE can relieve this condition to a certain extent.

## Conclusion

To predict the postoperative complication of pediatric cataracts, SMOTE is employed to preprocess dataset and then two types of classifier (random forest and naïve Bayes classifier) was exploited to classify samples with or without complication (two types of complication). All average accuracies in solving three binary classification problems are over 91%, and one is even reaching 95%. Then real factors that are more related to the complications were identified with genetic feature selection. Besides, the association rules that hide in the dataset can also provide some evidence about complication to assist doctors in treatment. Finally, additional 50 data were used to test the performance of RF and NB, and both the accuracies of them reach 65% for three classification problems. All these verified methods and models were integrated into a medical decision making system to help doctors to predict the postoperative complication of Pediatric Cataract Patients. In the future, more information, such as medication during pregnancy and genetic information, can be put into dataset to predict complications more accurately. As a type of rare disease, the data of pediatric cataracts is not enough. Therefore, we hope the on-line system in current research can assist in collecting more data and support the multicenter study in the future.

## Additional file

**Additional file 1: Table S1.** Association rules about whether a patient will have complications (*threshold* = 6). **Table S2.** Association rules about whether a patient will have the first type of complication (*threshold* = 7). **Table S3.** Association rules about whether a patient will have the second type of complication (*threshold* = 4).

## Abbreviations

RF: random forest
NB: Naïve Bayesian Classifier
ROC: receiver operating characteristics curve
AUC: area under curve
SMOTE: synthetic minority over sampling technique
