# Supplementary material for: Prediction of postoperative complications of pediatric cataract patients using data mining
Source: J Transl Med. 2019 Jan 3;17:2. doi: 10.1186/s12967-018-1758-2 (PMC6317183; doi:10.1186/s12967-018-1758-2)
Supplement: Supplementary file 1 — Additional file 1: Table S1. Association rules about whether a patient will have complications (threshold = 6). Table S2. Association rules about whether a patient will have the first type of complication (threshold = 7). Table S3. Association rules about whether a patient will have the second type of complication (threshold = 4). [file 12967_2018_1758_MOESM1_ESM.docx]

**Prediction of Postoperative Complications of Pediatric Cataract Patients using Data Mining**

Kai Zhang^1, 2^, Xiyang Liu^1, 3, 4§^, Jiewei Jiang^1, 2^, Wangting Li^2^, Shuai Wang^4^, Lin Liu^1^, Xiaojing Zhou^5^, Liming Wang^3, 4^

^1^School of Computer Science and Technology, Xidian University, Xi’an 710071, China;

^2^State Key Laboratory of Ophthalmology, Zhongshan Ophthalmic Center, Sun Yat-sen University, Guangzhou 510060, China;

^3^Institute of Software Engineering, Xidian University, Xi’an 710071, China;

^4^School of Software, Xidian University, Xi’an 710071, China;

^5^School of Computer Science, Northwestern Polytechnical University, Xi’an 710072, China;

^§^**Corresponding authors:**

Prof. Xiyang Liu, Email: xyliu@xidian.edu.cn; Address: Xidian University, No.2 South Taibai Rd, Xi’an, China, 710071. Telephone: +86-029-88204612; Fax: +86-029-88204612;

**Additional file 1: Table S1. Association rules about whether a patient will have complications ($threshold$= 6)**

| Antecedent | | Consequent | Confidence |
| --- | --- | --- | --- |
| Secondary IOL placement | Dense | Has | 0.111 |
|  |  | Has no | 0.888 |
| I/A | 1 | Has | 0.864 |
|  |  | Has no | 0.135 |
|  | Female | Has | 0.903 |
|  |  | Has no | 0.096 |
| Unilateral cataracts | 1 | Has | 0.886 |
|  |  | Has no | 0.113 |
|  | Sloppy | Has | 0.868 |
|  |  | Has no | 0.131 |
|  | Female | Has | 0.921 |
|  |  | Has no | 0.078 |
| 7 | Sloppy | Has | 0.864 |
|  |  | has no | 0.135 |
| Large | 1 | Has | 0.868 |
|  |  | Has no | 0.131 |
|  | Female | Has | 0.913 |
|  |  | Has no | 0.086 |
| Dense | 1 | Has | 0.883 |
|  |  | Has no | 0.116 |
|  | Female | Has | 0.897 |
|  |  | Has no | 0.102 |
| Cover the central area of lens | 1 | Has | 0.886 |
|  |  | Has no | 0.113 |
|  | Female | Has | 0.916 |
|  |  | Has no | 0.083 |
| Not nystagmus | 1 | Has | 0.878 |
|  |  | Has no | 0.121 |
|  | Female | Has | 0.905 |
|  |  | Has no | 0.094 |
| Not microphthalmia | 1 | Has | 0.875 |
|  |  | Has no | 0.125 |
|  | Female | Has | 0.913 |
|  |  | Has no | 0.086 |
| Not microcornea | 1 | Has | 0.880 |
|  |  | Has no | 0.119 |
|  | Female | Has | 0.916 |
|  |  | Has no | 0.083 |
| Not PHPV | 1 | Has | 0.886 |
|  |  | Has no | 0.113 |
|  | Female | Has | 0.919 |
|  |  | Has no | 0.080 |

(Note: 1, 2, 3, 4, 5, 6 and 7 stand for the seven age groups mentioned above; ‘Has no’ and ‘Has’ refer to not suffering from complications and suffering from complications)

**Additional file 1: Table S2. Association rules about whether a patient will have the first type of complication ($threshold$= 7)**

| Antecedent | | Consequent | Confidence |
| --- | --- | --- | --- |
| Female | Secondary IOL placement | Has | 0.076 |
|  |  | Has no | 0.923 |
|  | 7 | Has | 0.084 |
|  |  | Has no | 0.915 |
|  | Sloppy | Has | 0.084 |
|  |  | Has no | 0.915 |
| Secondary IOL placement | Large | Has | 0.097 |
|  |  | Has no | 0.902 |
|  | Sloppy | Has | 0.121 |
|  |  | Has no | 0.878 |
|  | Cover the central area of lens | Has | 0.097 |
|  |  | Has no | 0.902 |
|  | Nystagmus | Has | 0.117 |
|  |  | Has no | 0.882 |
|  | Not microphthalmia | Has | 0.121 |
|  |  | Has no | 0.878 |
|  | Not microcornea | Has | 0.121 |
|  |  | Has no | 0.878 |
| I/A | Primary IOL placement | Has | 0.909 |
|  |  | Has no | 0.090 |
| Unilateral cataracts |  | Has | 0.920 |
|  |  | Has no | 0.079 |
| Large | Sloppy | Has | 0.113 |
|  |  | Has no | 0.886 |
|  | Primary IOL placement | Has | 0.912 |
|  |  | has no | 0.087 |
| Sloppy | Cover the central area of lens | Has | 0.102 |
|  |  | Has no | 0.897 |
| Cover the central area of lens | Primary IOL placement | Has | 0.918 |
|  |  | Has no | 0.081 |
| Not microphthalmia |  | Has | 0.915 |
|  |  | Has no | 0.084 |
| Not microcornea |  | Has | 0.918 |
|  |  | Has no | 0.081 |
| Not PHPV |  | Has | 0.920 |
|  |  | Has no | 0.079 |

(Note: 1, 2, 3, 4, 5, 6 and 7 stand for the seven age groups mentioned above; ‘Has no’ and ‘Has’ refer to not suffering from the first complication and suffering from the first complication)

**Additional file 1: Table S3. Association rules about whether a patient will have the second type of complication ($threshold$= 4)**

| Antecedent | | Consequent | Confidence |
| --- | --- | --- | --- |
| Male | 5 | Has | 0.166 |
|  |  | Has no | 0.833 |
| Secondary IOL placement | Dense | Has | 0.141 |
|  |  | Has no | 0.858 |
|  | 1 | Has | 0.166 |
|  |  | Has no | 0.833 |
|  | 5 | Has | 0.166 |
|  |  | Has no | 0.833 |
| Unilateral cataracts | 1 | Has | 0.833 |
|  |  | Has no | 0.166 |
| 7 | Dense | Has | 0.176 |
|  |  | Has no | 0.823 |
| Large | 1 | Has | 0.807 |
|  |  | Has no | 0.192 |
| Dense |  | Has | 0.827 |
|  |  | Has no | 0.172 |
| Cover the central area of lens |  | Has | 0.833 |
|  |  | Has no | 0.166 |
| Not nystagmus |  | Has | 0.814 |
|  |  | Has no | 0.185 |
| Not microphthalmia |  | Has | 0.807 |
|  |  | Has no | 0.192 |
| Not microcornea |  | Has | 0.821 |
|  |  | Has no | 0.178 |
| Not PHPV |  | Has | 0.833 |
|  |  | Has no | 0.166 |

(Note: 1, 2, 3, 4, 5, 6 and 7 stand for the seven age groups mentioned above; ‘Has no’ and ‘Has’ refer to not suffering from the second complication and suffering from the second complication)
